# Urinary Metabolomic Approach Provides New Insights into Distinct Metabolic Profiles of Glutamine and *N*-Carbamylglutamate Supplementation in Rats

**DOI:** 10.3390/nu8080478

**Published:** 2016-08-04

**Authors:** Guangmang Liu, Wei Cao, Tingting Fang, Gang Jia, Hua Zhao, Xiaoling Chen, Caimei Wu, Jing Wang

**Affiliations:** 1 Institute of Animal Nutrition, Sichuan Agricultural University, Chengdu 611130, Sichuan, China; weicao_sicau@163.com (W.C.); fangtt132@126.com (T.F.); jiagang700510@163.com (G.J.); zhua666@126.com (H.Z.); xlchen@sicau.edu.cn (X.C.); zhuomuniao278@163.com (C.W.); 2 Key Laboratory for Animal Disease-Resistance Nutrition of China Ministry of Education, Chengdu 611130, Sichuan, China; 3 Maize Research Institute, Sichuan Agricultural University, Chengdu 611130, Sichuan, China; wangj221@gmail.com

**Keywords:** glutamine, *N*-carbamylglutamate, metabolism, metabolomics, urine

## Abstract

Glutamine and *N*-carbamylglutamate can enhance growth performance and health in animals, but the underlying mechanisms are not yet elucidated. This study aimed to investigate the effect of glutamine and *N*-carbamylglutamate supplementation in rat metabolism. Thirty rats were fed a control, glutamine, or *N*-carbamylglutamate diet for four weeks. Urine samples were analyzed by nuclear magnetic resonance (NMR)-based metabolomics, specifically high-resolution ^1^H NMR metabolic profiling combined with multivariate data analysis. Glutamine significantly increased the urine levels of acetamide, acetate, citrulline, creatinine, and methymalonate, and decreased the urine levels of ethanol and formate (*p* < 0.05). Moreover, *N*-carbamylglutamate significantly increased the urine levels of creatinine, ethanol, indoxyl sulfate, lactate, methymalonate, acetoacetate, *m*-hydroxyphenylacetate, and sarcosine, and decreased the urine levels of acetamide, acetate, citrulline, creatine, glycine, hippurate, homogentisate, *N*-acetylglutamate, phenylacetyglycine, acetone, and *p*-hydroxyphenylacetate (*p* < 0.05). Results suggested that glutamine and *N*-carbamylglutamate could modify urinary metabolome related to nitrogen metabolism and gut microbiota metabolism. Moreover, *N*-carbamylglutamate could alter energy and lipid metabolism. These findings indicate that different arginine precursors may lead to differences in the biofluid profile in rats.

## 1. Introduction

Glutamine, a conditionally essential amino acid under inflammatory and many other stress conditions in both humans and animals [1,2], can increase cellular adenosine triphosphate levels [3,4]. Approximately 70% of glutamine in the enteral diet is degraded by rat and pig small intestines during the first pass and becomes a primary fuel for the growth and renewal of intestinal cells [5,6]. Glutamine can also regulate intestinal gene expression [7], protect intestinal epithelial tight junctions, increase the absorption and utilization of nutrients and immune function in animal production worldwide [8,9], modulate intracellular protein turnover [10], prevent intestinal oxidative injury, and inhibit cell autophagy [11]. Glutamine can also increase mitochondrial function [12]. Thus, glutamine is an important regulator for maintaining intestinal barrier integrity and function. Moreover, glutamine can enhance growth performance of early-weaned piglets [7]. Furthermore, glutamine supplementation between 90 and 114 days of gestation ameliorates fetal growth restriction in gilts and decreases pre-weaning mortality of piglets [1]. Finally, glutamine can increase milk production by lactating sows and survival of suckling piglets [1].

*N*-carbamylglutamate, a metabolically-stable analogue of *N*-acetylglutamate that activates intestinal pyrroline-5-carboxylate synthase and carbamylphosphate synthase-1 (key enzymes in arginine synthesis in enterocytes), provides a novel, effective strategy to increase endogenous arginine provision and can treat hyperammonaemia [13,14,15]. *N*-carbamylglutamate has been reported as having no toxicity for animals or humans [16], and it has been shown to stimulate citrulline synthesis in enterocytes and increase plasma concentrations of arginine and somatotropin [13,17]. *N*-carbamylglutamate administration increases absolute rates of muscle protein synthesis and growth rate in sow-reared piglets and improves the litter size in animals [13,18]. Furthermore, dietary *N*-carbamylglutamate supplementation enhance intestinal growth, and improve intestinal function in weaned pigs [19]. However, the exact mechanisms by which glutamine and *N*-carbamylglutamate contribute to various health conditions remain unclear. Therefore, the health effects of glutamine and *N*-carbamylglutamate supplementation and knowledge of these mechanisms need to be elucidated.

Recent metabolomics studies revealed that glutamine supplementation can affect the plasma metabolome in pigs [20], and *N*-carbamylglutamate can alter biofluid metabolome of rats under oxidative stress conditions [21]. However, no information has focused on the response of animal or human urinary biological systems to glutamine supplementation. No studies are available on the response of animal or human biological systems to *N*-carbamylglutamate under normal condition. Moreover, there is no information about the difference of metabolic profiles between glutamine and *N*-carbamylglutamate in any mammalian in vivo system. Metabolomics provides a novel strategy to resolve the changes in metabolic endpoints of physiological regulatory processes of an organism after the administration of specific nutritional interventions. Metabolomics is potentially valuable to the study of glutamine and *N*-carbamylglutamate metabolism and the search for relationships between glutamine and *N*-carbamylglutamate supplementation and health and disease. This experiment aimed to examine the effects of glutamine and *N*-carbamylglutamate supplementation on the urinary compositions of rats using an explorative metabolomic approach through proton nuclear magnetic resonance (^1^H NMR) spectroscopy and chemometrics.

## 2. Materials and Methods

### 2.1. Animal Experiment and Sample Collection

The animal experiment was approved by the Animal Care and Use Committee of Sichuan Agricultural University. It was performed according to the Guide for the Care and Use of Laboratory Animals of the National Research Council. A total of 30 eight-week-old female Sprague–Dawley rats weighing 249 g to 277 g were placed in individual metabolic cages and allowed to acclimatize for two weeks. After this period, the rats were assigned randomly to three purified dietary groups, with 10 rats in each group, for 28 days. The rats were fed with a basal diet containing 0 (control), 1% glutamine (supplied by Beijing Jiakangyuan Technology Development Co., Ltd., Beijing, China), or 0.1% *N*-carbamylglutamate (supplied by Asia Pacific Xingmu Technology Co., Ltd., Beijing, China). Urine samples were collected in ice-cooled vessels, including 30 µL of sodium azide solution (1.0% w/v) from day 27 to day 28 of the treatment period (24 h). All urine samples were stored at −80 °C until NMR analysis was performed. Rats were allowed free access to food and drinking water. Temperatures between 22 °C and 25 °C, a cycle of 12 h light/12 h dark, and humidity ranging from 50% to 70% were maintained throughout the duration of the study. Clinical observations were conducted during the whole experimental period. The dosage selected for this study was based on the results of a previous experiment [7,17,19].

### 2.2. Sample Preparation and NMR Spectroscopy

Urine samples (550 µL) were mixed with 55 µL of phosphate buffer (1.5 M NaH_2_PO_4_/K_2_HPO_4_, pH 7.4, 100% v/v D_2_O) containing 0.1% NaN_3_ as bacterial growth inhibitor and 5.0 mM 2,2-dimethyl-2-silapentane-5-sulfonate-d_6_ (DSS) as chemical shift reference (δ0.00 ppm). The supernatant was transferred into 5 mm NMR tubes for the NMR test after vortex mixing and 10 min of centrifugation (4 °C) at 12,000 *g*.

The proton NMR spectra of the urine samples were recorded at 300 K on a Bruker Avance II 600 MHz spectrometer (600.13 MHz for ^1^H frequency; Bruker Biospin, Rheinstetten, Germany) with a broadband-observe probe. A standard water-suppressed 1D NMR spectrum was obtained from urine using the first increment of the gradient-selected NOESY pulse sequence (recycle delay–90°–*t*_1_–90°–*t*_m_–90°–acquire data) with recycle delay of 2 s, *t*_1_ of 3 µs, mixing time (*t*_m_) of 100 ms, and 90° pulse length of 13.70 µs. A total of 128 transients were collected into 49,178 data points using a spectral width of 9590 Hz and an acquisition time of 2.56 s. Metabolites were generally assigned by considering the chemical shifts, coupling constants, and relative intensities, as in previous reports [22,23,24], and additional ^1^H–^1^H correlation spectroscopy and ^1^H–^1^H total correlation spectroscopy were recorded for selected samples (data not shown).

### 2.3. NMR Spectroscopic Processes and Analysis

Prior to Fourier transformation, the free induction decays were multiplied by an exponential function with a line-broadening factor of 1 Hz. All ^1^H NMR spectra were manually corrected for phase and baseline distortions by Mestrenova 8.1.2 software (Mestrelab Research S.L., Santiago de Compostela, Spain). The urinary spectral region δ0.5 to δ9.5 was binned with an equal width of 0.005 ppm using Mestrenova 8.1.2 software (Mestrelab Research S.L., Santiago de Compostela, Spain). Urine chemical shifts were referenced to the peak of DSS at δ0.00. Chemical shifts for urinary citrate were manually corrected because its signals had large inter-sample variations. To obtain the endogenous metabolite changes induced by treatment, the regions in the urine spectra containing δ4.50 to δ5.30 for H_2_O, as well as δ5.5 to δ6.0 for urea, were excluded. To compensate for the differences in concentration between different samples, urine spectra was normalized to the total sum of all integral regions for each spectrum before pattern recognition analysis.

Multivariate data analysis was achieved on normalized NMR datasets with the software package SIMCA-P+ (version 11.0, Umetrics, Umeå, Sweden). Principal component analysis (PCA) of the ^1^H NMR spectral data was performed on the mean-centered data to visualize the general structure of each dataset and identify any abnormalities (based on the principles of Hotelling’s T^2^) within the dataset. Results were observed in the form of score plots, in which each point represented an individual sample, and loading plots, in which each coordinate represented one NMR spectral region. Projection to latent structure-discriminant analysis (PLS-DA) and orthogonal projection to latent structure-discriminant analysis (OPLS-DA) were applied to the analysis of ^1^H NMR spectral data scaled to unit variance as the X-matrix and the class information as the Y-matrix to uncover metabolic differences [24]. The quality of the model was evaluated by such model parameters as R^2^X, which indicates the total explained variation, and Q^2^, which represents the model predictability. The models were validated using two methods: a seven-fold cross-validation method and a permutation test [25,26]. In this study, appropriate correlation coefficients greater than the cutoff values (depending on the sample numbers of animals in each group) were considered to be statistically significant (*p* < 0.05). The coefficients were determined by Pearson’s product-moment correlation coefficient.

## 3. Results

### 3.1. ^1^H NMR Spectra of Urine Samples

**Figure 1:**
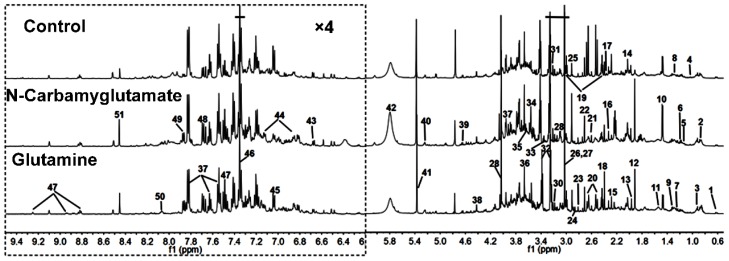
Representative 1D ^1^H NMR spectra urine metabolites obtained from the control, *N*-carbamylglutamate, and glutamine groups. The region of δ6.2–9.5 was magnified four times compared with the corresponding region of δ0.5–6.2 for the purpose of clarity. Metabolite keys are given in Table 1.

Figure 1 demonstrates typical ^1^H NMR spectra of the urine samples obtain from randomly selected rats in the glutamine, *N*-carbamylglutamate, and control groups. NMR signals were distributed to specific metabolites for ^1^H resonances (Table 1). Fifty-one metabolites were assigned to urine. The spectra of the urine samples included resonances from several amino acids and organic acids, as well as glucose, allantoin, and choline. Tricarboxylic acid cycle metabolites, such as succinate, α-ketoglutarate, and citrate, were also detected in the urine samples. 

### 3.2. Multivariate Data Analysis of NMR Data

**Figure 2:**
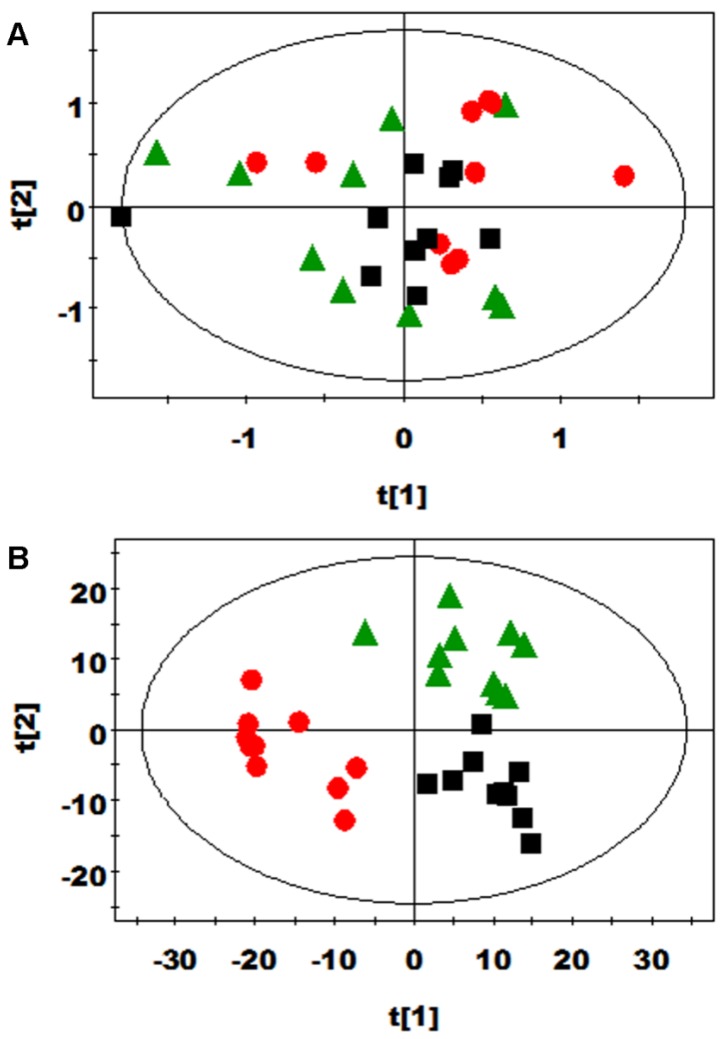
(**A**) PCA (R2X = 0. 545, Q2 = 0.06) and (**B**) PLS-DA score plots (R2X = 0.192, R2Y = 0.795, Q2 = 0.474) based on the ^1^H NMR spectra of the urine obtained from urinary metabolites from the control (black squares), glutamine (green triangles), and *N*-carbamylglutamate (red circles) groups.

**Figure 3:**
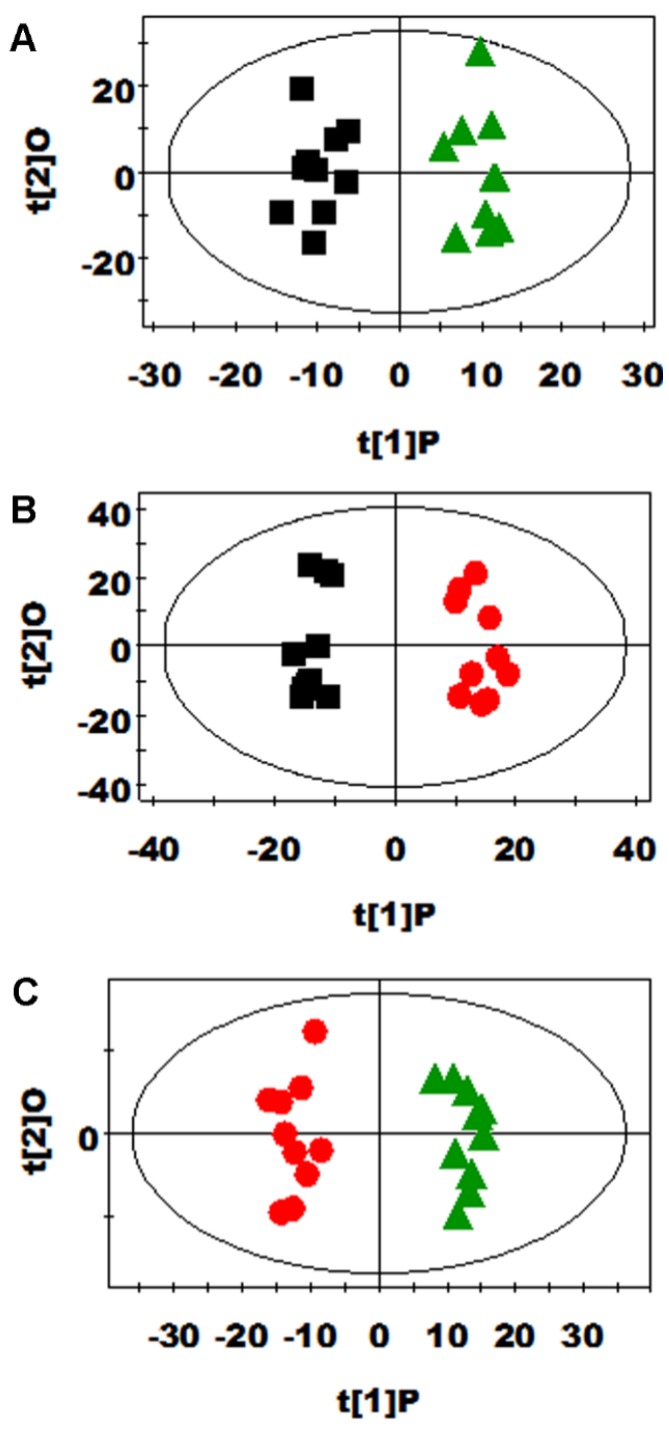
OPLS-DA score plots of urinary metabolites derived from the control (black squares), glutamine (green triangles), and *N*-carbamylglutamate (red circles) ((**A**), R2X = 0.202, Q2 = 0.486; (**B**), R2X = 0.307, Q2 = 0.773; (**C**), R2X = 0.292, Q2 = 0.780) groups.

**Figure 4:**
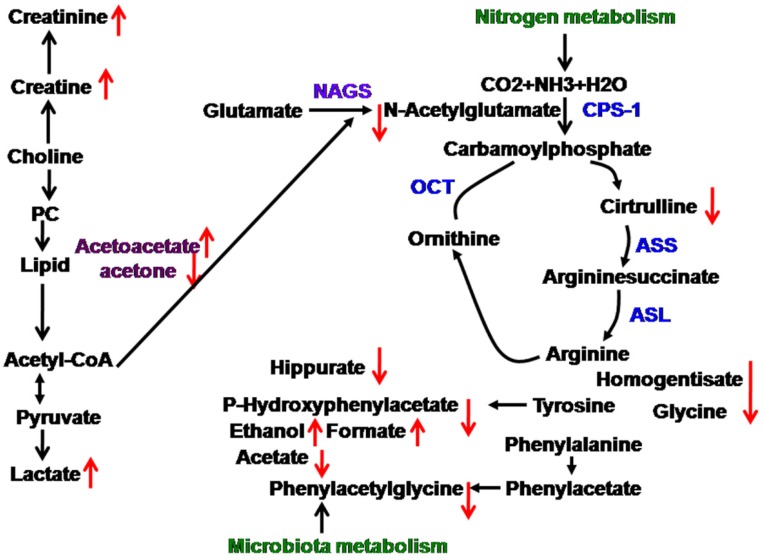
*N*-carbamylglutamate-induced changes in the metabolic pathway, 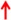 upregulated, 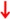 downegulated; PC, phosphorylcholine; NAGS, *N*-acetylglutamate; CPS-1, carbamoylphosphate synthetase-1; ASS, argininosuccinate synthase; ASL, argininosuccinate lyase; OCT, ornithine carbamonyltransferase.

PCA and PLS-DA were initially carried out on the urinary spectral data (Figure 2). Two principal components were calculated for the treatment groups, with 19.7% and 17.6% of the variables being explained by PC1 and PC2, respectively. PCA results (Figure 2A) showed that the separations in rats from the glutamine, *N*-carbamylglutamate, and control groups were absent in their metabolic urinary profiles. PLS-DA was conducted on the urine spectra of the glutamine, *N*-carbamylglutamate, and control groups. The score plots (Figure 2B) highlighted three clusters corresponding to the three groups. The metabolic profiles of the glutamine, *N*-carbamylglutamate, and control groups were compared using OPLS-DA to further identify the important urine metabolic changes induced by amino acid supplementation. Multivariate data analysis showed that the urine levels of acetamide, acetate, citrulline, creatinine, and methymalonate were higher in the glutamine group than those in the control group (*p* < 0.05). By contrast, the urine levels of ethanol and formate were lower in the glutamine group than in the control group (*p* < 0.05, Figure 3A and Table 2). The metabolic profile of the *N*-carbamylglutamate group was compared with that of the control group using OPLS-DA to observe the effect of *N*-carbamylglutamate supplementation. The urine levels of creatinine, ethanol, indoxyl sulfate, lactate, methymalonate, acetoacetate, *m*-hydroxyphenylacetate, and sarcosine were significantly higher in the *N*-carbamylglutamate group than in the control group (*p* < 0.05). By contrast, the urine levels of acetamide, acetate, citrulline, creatine, glycine, hippurate, homogentisate, *N*-acetylglutamate, phenylacetyglycine, acetone, and *p*-hydroxyphenylacetate were lower in the *N*-carbamylglutamate group than those in the control group (*p* < 0.05, Figure 3B and Figure 4 and Table 2). OPLS-DA was also carried out to determine the degree of influence of glutamine supplementation on metabolism compared with the *N*-carbamylglutamate group. The urine levels of acetamide, acetate, citrulline, creatine, hippurate, homogentisate, *N*-acetylglutamate, acetone, and *p*-hydroxyphenylacetate were significantly higher in the glutamine group than those in the *N*-carbamylglutamate group (*p* < 0.05). By contrast, the urine levels of ethanol, indoxyl sulfate, methymalonate, α-ketoglutarate, acetoacetate, *m*-hydroxyphenylacetate, sarcosine, pyruvate, and methylamine were lower those in the glutamine group than those in the *N*-carbamylglutamate group (*p* < 0.05, Figure 3C and Table 2).

## 4. Discussion

Both glutamine and *N*-carbamylglutamate are the important precursors of arginine synthesis, can regulate body metabolism, and increase growth performance in animals. However, the knowledge of their exact mechanisms of action remain largely understood. In this experiment, the daily bodyweight gain of glutamine and *N*-carbamylglutamate were higher than that of the contol group, however, the daily bodyweight gain between glutamine and *N*-carbamylglutamate was not significant in statistics (data not shown). Thus, glutamine and *N*-carbamylglutamate can enhance growth performance in rats. The growth performance is related with body metabolism modulated by glutamine and *N*-carbamylglutamate supplementation. The effects of nutrients in the metabolic profiles are more evident in the urine than in the plasma [27]. The collection of urine is noninvasive. Thus, a urinary metabolomic approach was used.

*N*-carbamylglutamate supplementation could affect lipid metabolism. Ketone body production (such as acetone, 3-hydroxybutyrate, and acetoacetate) provides fuel for vital organs (such as the heart and brain), thus increasing the chance of survival from metabolic problems. In the current study, acetoacetate significantly improved, but acetone decreased. This is not in agreement with the result of a previous study: *N*-carbamylglutamate supplementation can significantly increase plasma and urine acetone content, but exerted no effect on urine and plasma acetoacetate under oxidative stress [21]. The reason for this difference is unclear and needs further investigation in the future. Collectively, lipid metabolism was altered in rats. Moreover, *N*-carbamylglutamate supplementation could alter energy metabolism in rats. The *N*-carbamylglutamate group exhibited increased urinary lactate concentrations. Lactate is the end product of compounds associated with energy metabolism. The increase in lactate may imply carbohydrate and energy metabolism modification. This is not consistent with the result of our previous study [21]. The urinary creatine levels increased in the *N*-carbamylglutamate group compared with those in the control group. Creatine provides energy to muscles of vertebrates in the form of stored creatine phosphate. Creatine levels in the animals are synthesized de novo from the liver via the use of amino acids, such as arginine, glycine, and methionine. This is in agreement with the result of previous study: *N*-carbamylglutamate supplementation can increase arginine concentration [13]. While arginine is a substrate for the synthesis of creatine. This is also consistent with the result of increased creatinine levels. Enhanced creatinine levels were also observed to be associated with growth. Creatinine is an index of muscle mass, which also supported our findings. To our knowledge, this is the first report regarding the difference of acetoacetate and lactate metabolites for *N*-carbamylglutamate supplementation under normal condition. Collectively, *N*-carbamylglutamate supplementation could modify energy metabolism in rats.

Glutamine and *N*-carbamylglutamate supplementation can alter nitrogen metabolism. Glutamine and *N*-carbamylglutamate are known to have important functions in increasing protein synthesis, which results in the conversion of more amino acids into proteins. Glutamine increased urinary citrulline. Citrulline is an amino acid made from ornithine and carbamoyl phosphate in one of the central reactions in the urea cycle. Citrulline is derived from arginine as a byproduct of the reaction catalyzed by the nitric oxide synthase family. In this reaction, arginine is first oxidized into *N*-hydroxyl-arginine and then further oxidized to citrulline together with the release of nitric oxide [28]. This was in agreement with the result of a previous study [29]; thus, glutamine can increase plasma citrulline. This increase may be caused by increased intestinal citrulline [24]. *N*-carbamylglutamate decreased urinary *N*-acetylglutamate levels. This was in accordance with the result of a previous study [29]. However, *N*-carbamylglutamate decreased urinary citrulline level. This was different from the result of a previous study, which found that *N*-carbamylglutamate can increase plasma citrulline in piglets [30]. The reason for this difference is unclear and needs further investigation in the future. Urea has a crucial function in the metabolism of nitrogen-containing compounds. *N*-acetylglutamate is needed for the normal function of the urea cycle, and variations in *N*-acetylglutamate concentrations modify the urea production rate and other substrates for urea synthesis [31]. Furthermore, homogentisate is an intermediate of the metabolic breakdown of tyrosine and phenylalanine [32]. Sarcosine is the *N*-methyl derivative of glycine. In the present study, homogentisate and glycine concentrations significantly decreased, but sarcosine concentrations increased by *N*-carbamylglutamate supplementation. These were not in line with the results of a previous study [21]. The reason for this difference is unclear and needs further investigation in the future. To our knowledge, this is the first report regarding the difference of homogentisate and sarcosine metabolites for *N*-carbamylglutamate supplementation under normal condition. Therefore, glutamine and *N*-carbamylglutamate supplementation could alter nitrogen metabolism in rats.

Glutamine and *N*-carbamylglutamate can change gut microbiota functions. In this study, urinary formate, acetate, and ethanol concentrations were affected by glutamine supplementation. Urinary formate and ethanol concentrations were also affected by *N*-carbamylglutamate. Notably, urinary formate and ethanol are microbial metabolites of carbohydrates, which are likely produced in the lumen of the small and large intestines [33]. Changes in these metabolites may result from altered activity of intestinal microorganisms. Moreover, the urine level of microbiotic metabolites such as *m*-hydroxyphenylacetate significantly increased. This was not in accordance with the result of a previous study [21]. The possible reason is that oxidative stress may affect the microbiotic metabolism and show the difference of metabolites compared with non-oxidative stress condition, which demonstrates no change of *m*-hydroxyphenylacetate in oxidative stress condition and the increase of *m*-hydroxyphenylacetate in non-oxidative stress. Furthermore, results of this study also indicated that *N*-carbamylglutamate decreased the urinary excretion of hippurate, which is produced via both renal and hepatic syntheses of benzoic acid and glycine. This was consistent with the result of our previous study [21]. Hippurate is considered as the degradation product of flavonols acted upon by intestinal microorganisms [34]. As a result, a change in the excretion of this compound suggests a corresponding change in the functional metabolism of the microbiota. Variations in urinary hippurate concentration have also been associated with the changes in the distribution of intestinal microbial colonies [35]. Changes in gut microbial co-metabolites, such as phenylacetylglycine and *p*-hydroxyphenylacetate, with *N*-carbamylglutamate exposure, verified the association of the disturbance to gut microbiota. Through the action of gut microbiota, phenylacetate was transformed from phenylalanine through the action of gut microbiota, and phenylacetate was then conjugated with glycine to produce phenylacetylglycine [35]. *p*-Hydroxyphenylacetate is a metabolite of tyrosine via the action of enteric bacteria. These were not in line with the results of previous study [21]. The possible reason is that oxidative stress may affect the microbiotic metabolism and show the difference of metabolites compared with non-oxidative stress conditions. In the current study, glutamine supplementation significantly increased the urinary acetamide levels. However, *N*-carbamylglutamate decreased urinary acetamide. Acetamide exhibits anti-microbial, anti-inflammatory, anti-arthritic, and antibiotic functions [36,37]. Changes in these metabolites are attributed to the altered activity of intestinal microorganisms. Glutamine increased the concentrations of urinary acetate and acetamide, whereas *N*-carbamylglutamate decreased the concentrations of urinary acetate, acetamide, hippurate, phenylacetyglycine, and *p*-hydroxyphenylacetate. These findings were possibly due to the differences of arginine precursor sources. To our knowledge, this is the first report regarding the difference of formate, ethanol, *m*-hydroxyphenylacetate, *p*-hydroxyphenylacetate, hippurate, and acetamide metabolites for *N*-carbamylglutamate supplementation under normal condition. Mammalian metabolism is greatly affected by the complex gut microbiota [38]. The introduction of glutamine and *N*-carbamylglutamate into the mammalian system may displace baseline mammalian-to-microbial behavior, thereby disrupting microbial populations and eventually affecting metabolism.

## 5. Conclusions

Glutamine and *N*-carbamylglutamate can alter some common systemic metabolic processes, including nitrogen and gut microbiota metabolism. Moreover, *N*-carbamylglutamate can alter energy and lipid metabolism. These results indicate that different arginine precursor may cause differences in the biofluid profile in rats. This research contributes in defining the effects of metabolic modifiers to offer better nutritional support for growth and health. This study emphasized the potential metabolomic strategy in the evaluation of nutritional interventions in a mammalian system. To the best of our knowledge, this study is the first to systematically identify the distinct urinary metabolic profiles between glutamine and *N*-carbamylglutamate supplementation.

## Figures and Tables

**Table 1:** Proton nuclear magnetic resonance (^1^H NMR) data of metabolites in rat urine.

Keys	Metabolites	Moieties	δ ^1^H (ppm) and Multiplicity	Samples
1	Bile acids	CH_3_	0.64(m), 0.75(m)	U
2	α-Hydroxy-iso-valerate	δCH_3_, CH_3_	0.83(d), 0.97(d)	U
3	α-Hydroxybutyrate	CH_3_	0.89(t)	U
4	Propionate	CH_3_	1.06(t)	U
5	Isobutyrate	CH_3_	1.13(d)	U
6	Ethanol	CH_3_	1.19(t)	U
7	Methylmalonate	CH_3_, CH	1.25(d), 3.75(m)	U
8	α-Hydroxy-n-valerate	CH_3_, γCH_2_	0.89(t), 1.31(m)	U
9	Lactate	αCH, βCH_3_	4.14(q), 1.33(d)	U
10	Alanine	αCH, βCH_3_	3.77(q), 1.47(d)	U
11	Citrulline	γCH_2_, βCH_2_	1.56(m), 1.82(m)	U
12	Acetate	CH_3_	1.92(s)	U
13	Acetamide	CH_3_	1.99(s)	U
14	*N*-Acetylglutamate	βCH_2_, γCH_2_, CH_3_	2.06(m), 1.87(m), 2.03(s)	U
15	Acetone	CH_3_	2.24(s)	U
16	Acetoacetate	CH_3_	2.28(s)	U
17	Pyruvate	CH_3_	2.33(s)	U
18	Succinate	CH_2_	2.40(s)	U
19	α-Ketoglutarate	βCH_2_, γCH_2_	2.45(t), 3.01(t)	U
20	Citrate	CH_2_	2.54(d), 2.68(d)	U
21	Methylamine	CH_3_	2.61(s)	U
22	Dimethylamine	CH_3_	2.71(s)	U
23	Methylguanidine	CH_3_	2.81(s)	U
24	Trimethylamine	CH_3_	2.88(s)	U
25	Dimethylglycine	CH_3_	2.93(s)	U
26	Creatine	CH_3_, CH_2_	3.04(s), 3.93(s)	U
27	Creatinine	CH_3_, CH_2_	3.04(s), 4.05(s)	U
28	Ornithine	CH_2_	3.06(t)	U
29	Ethanolamine	CH_2_	3.11(t)	U
30	Malonate	CH_2_	3.15(s)	U
31	Choline	OCH_2_, NCH_2_, N(CH_3_)_3_	4.07(t), 3.53(t), 3.21(s)	U
32	Taurine	–CH_2_-S, –CH_2_–NH_2_	3.27(t), 3.43(t)	U
33	TMAO ^a^	CH_3_	3.27(s)	U
34	Glycine	CH_2_	3.57(s)	U
35	Sarcosine	CH_2_	3.6(s)	U
36	Phenylacetyglycine	2,6–CH, 3,5–CH, 7–CH, 10–CH	7.30(t), 7.36(m), 7.42(m), 3.67(s)	U
37	Hippurate	CH_2_, 3,5–CH, 4–CH, 2,6–CH	3.97(d), 7.55(t), 7.63(t), 7.84(d)	U
38	*N*-Methylnicotinamide	CH_3_, 5–CH, 4–CH, 6–CH, CH_2_	4.42(s), 8.21(d), 8.87(d), 8.93(d), 9.24(s)	U
39	β-Glucose	1–CH, 2–CH, 3–CH, 4–CH, 5–CH, 6–CH	4.47(d), 3.25(dd), 3.49(t), 3.41(dd), 3.46(m), 3.73(dd), 3.90(dd)	U
40	α-Glucose	1–CH, 2–CH, 3–CH, 4–CH, 5–CH, 6–CH	5.24(d), 3.54(dd), 3.71(dd), 3.42(dd), 3.84(m), 3.78(m)	U
41	Allantoin	CH	5.39(s)	U
42	Urea	NH_2_	5.82(s)	U
43	Homogentisate	6–CH, 5–CH	6.67(d), 6.82(d)	U
44	*p*-Hydroxyphenylacetate	6–CH, 2–CH, 3,5–CH	3.6(s), 6.85(d), 7.15(d)	U
45	*m*-Hydroxyphenylacetate	6–CH, 4–CH, 3–CH	6.92(m), 7.04(d), 7.26(t)	U
46	Indoxyl sulfate	4–CH, 5–CH, 6–CH, 7–CH, CH	7.51(m), 7.22(m), 7.28(m), 7.71(m), 7.37(s)	U
47	Nicotinate	2,6–CH, 4–CH, 5–CH	8.60(d), 8.25(d), 7.5(dd)	U
48	4-Aminohippurate	CH_2_, CH	7.6(d), 6.8(d), 3.9(d)	U
49	Benzoate	2,6–CH, 3,5–CH, 4–CH	7.87(d), 7.49(dd), 7.56(t)	U
50	Trigonelline	2–CH, 4–CH, 6–CH, 5–CH, CH3	9.09(s), 8.85(m), 8.81(dd), 8.07(m), 4.44(s)	U
51	Formate	CH	8.46(s)	U

^a^ TMAO, trimethylamine-*N*-oxide; s, singlet; d, doublet; t, triplet; q, quartet; dd, doublet of doublets; m, multiplet.

**Table 2:** Orthogonal projection to latent structure-discriminant analysis (OPLS-DA) coefficients derived from the NMR data of urine metabolites obtained from the (A) control, (B) glutamine, and (C) *N*-carbamylglutamate groups.

Metabolite	B (vs. A) ^a^	C (vs. A) ^a^	B (vs. C) ^a^
Acetamide (13)	0.608	−0.728	0.906
Acetate (12)	0.713	−0.742	0.768
Citrulline (11)	0.758	−0.962	0.966
Creatine (26)	—	−0.790	0.783
Creatinine (27)	0.723	0.717	—
Ethanol (6)	−0.630	0.692	−0.631
Formate (51)	−0.621	—	—
Glycine (34)	—	−0.616	—
Hippurate (37)	—	−0.914	0.906
Homogentisate (43)	—	−0.810	0.834
Indoxyl sulfate (46)	—	0.786	−0.786
Lactate (9)	—	0.653	—
Methylmalonate (7)	0.738	0.608	−0.653
*N*-Acetylglutamate (14)	—	−0.967	0.978
Phenylacetyglycine (36)	—	−0.634	—
α-Hydroxy-n-valerate (8)	−0.684	—	—
α-Ketoglutarate (19)	—	—	−0.623
Acetoacetate (16)	—	0.786	−0.815
Acetone (15)	—	−0.912	0.944
*m*-Hydroxyphenylacetate (45)	—	0.815	−0.883
*p*-Hydroxyphenylacetate (44)	—	−0.813	0.844
Sarcosine (35)	—	0.865	−0.853
α-Hydroxy-iso-valerate (2)	—	0.607	—
Pyruvate (4)	—	—	−0.608
Methylamine (21)	—	—	−0.635

^a^ Correlation coefficients: positive and negative signs indicate positive and negative correlations in the concentrations, respectively. The correlation coefficient of |*r*|> 0.602 was used as the cutoff value. ‘‘—’’ means the correlation coefficient |*r*| is less than 0.602. Analysis of relative integral from metabolites was given in Table S1 (Supplementary Material).

## Supplementary Materials

The following are available online at http://www.mdpi.com/2072-6643/8/8/478/s1, Table S1: Orthogonal projection to latent structure-discriminant analysis (OPLS-DA) coefficients derived from the NMR data of urine metabolites obtained from the (A) control, (B) glutamine, and (C) *N*-carbamylglutamate groups.
