# Supplementary material for: Urinary Metabolomic Approach Provides New Insights into Distinct Metabolic Profiles of Glutamine and N-Carbamylglutamate Supplementation in Rats
Source: Nutrients. 2016 Aug 4;8(8):478. doi: 10.3390/nu8080478 (PMC4997391; doi:10.3390/nu8080478)
Supplement: Supplementary file 1 [file nutrients-08-00478-s001.docx]

Supplemental Materials: Urinary Metabolomic Approach Provides New Insights into Distinct Metabolic Profiles of Glutamine and
*N*-Carbamylglutamate Supplementation in Rats

Guangmang Liu, Wei Cao, Tingting Fang, Gang Jia, Hua Zhao, Xiaoling Chen, Caimei Wu and Jing Wang

**Table S1.** Orthogonal projection to latent structure-discriminant analysis (OPLS‐DA) coefficients derived from the NMR data of urine metabolites obtained from the (A) control, (B) glutamine, and (C) *N*-carbamylglutamate groups.

| **Metabolite** | **OPLS-DA Coefficient (*r*) ^a^** | | | ***p* Value ^b^** | | |
| --- | --- | --- | --- | --- | --- | --- |
|  | **B (vs. A)** | **C (vs. A)** | **B (vs. C)** | **B (vs. A)** | **C (vs. A)** | **B (vs. C)** |
| Acetamide (13) | 0.608 | −0.728 | 0.906 | <0.05 | <0.05 | <0.05 |
| Acetate (12) | 0.713 | −0.742 | 0.768 | <0.05 | <0.05 | <0.05 |
| Citrulline (11) | 0.758 | −0.962 | 0.966 | <0.05 | <0.05 | <0.05 |
| Creatine (26) | – | −0.790 | 0.783 | >0.05 | <0.05 | <0.05 |
| Creatinine (27) | 0.723 | 0.717 | – | <0.05 | <0.05 | >0.05 |
| Ethanol (6) | −0.63 | 0.692 | −0.631 | <0.05 | <0.05 | <0.05 |
| Formate (51) | −0.621 | – | – | <0.05 | >0.05 | >0.05 |
| Glycine (34) | – | −0.616 | – | >0.05 | <0.05 | >0.05 |
| Hippurate (37) | – | −0.914 | 0.906 | >0.05 | <0.05 | <0.05 |
| Homogentisate (43) | – | −0.810 | 0.834 | >0.05 | <0.05 | <0.05 |
| Indoxyl sulfate (46) | – | 0.786 | −0.786 | >0.05 | <0.05 | <0.05 |
| Lactate (9) | – | 0.653 | – | >0.05 | <0.05 | >0.05 |
| Methymalonate (7) | 0.738 | 0.608 | −0.653 | <0.05 | <0.05 | <0.05 |
| *N*-Acetylglutamate (14) | – | −0.967 | 0.978 | >0.05 | <0.05 | <0.05 |
| Phenylacetyglycine (36) | – | −0.634 | – | >0.05 | <0.05 | >0.05 |
| α-Hydroxy-n-valerate (8) | −0.684 | – | – | <0.05 | >0.05 | >0.05 |
| α-Ketoglutarate(19) | – | – | −0.623 | >0.05 | >0.05 | <0.05 |
| Acetoacetate (16) | – | 0.786 | −0.815 | >0.05 | <0.05 | <0.05 |
| Acetone (15) | – | −0.912 | 0.944 | >0.05 | <0.05 | <0.05 |
| *m*-Hydroxyphenylacetate (45) | – | 0.815 | −0.883 | >0.05 | <0.05 | <0.05 |
| *p*-Hydroxyphenylacetate (44) | – | −0.813 | 0.844 | >0.05 | <0.05 | <0.05 |
| Sarcosine (35) | – | 0.865 | −0.853 | >0.05 | <0.05 | <0.05 |
| α-Hydroxy-iso-valerate (2) | – | 0.607 | – | >0.05 | <0.05 | >0.05 |
| Pyruvate (4) | – | – | −0.608 | >0.05 | >0.05 | <0.05 |
| Methylamine (21) | – | – | −0.635 | >0.05 | >0.05 | <0.05 |

^a^ Correlation coefficients: positive and negative signs show positive and negative correlation in the concentrations, respectively. The correlation coefficient of |*r*|> 0.602 was used as the cutoff value.
‘‘–’’ means the correlation coefficient |*r*| is less than 0.602; ^b^ Normalized integral of metabolites in the spectrum (normalized to 100). Integrals of the altered metabolites were analyzed statistically using one-way analysis of variance (ANOVA) of SPSS 16.0 software (SPSS Inc., Chicago, IL, USA). Datasets were further analyzed using post hoc tests (least significant difference, LSD) for multiple comparisons to determine the statistical differences between groups. *p* values are significant at the < 0.05 level, and *p* values are not significant at > 0.05.
